# The Amalgam Question

**Published:** 1885-12

**Authors:** J. Hardman

**Affiliations:** Muscatine, Iowa.


ARTICLE IV.

### THE AMALGAM QUESTION.

BY J. HARDMAN, OF MUSCATINE, IOWA.

Within the limits of a magazine article, it is impossible to use detail, or much tabulated formula upon such an extensive question as the manufacture of alloy for dental purposes, and the modes of using the same as amalgam in the treatment of carious teeth, etc.

I will at present merely attempt to offer some points for consideration seeming to have at this time, more or less especial interest to the practicing dentist. For the reader wishing fuller detail, and at the same time, the best obtainable authority, I can, with confidence, recommend Prof. J. Foster Flagg's work on "Plastics and Plastic Fillings," issued in 1881. In this work he will find much valuable information; being the most complete work upon plastic agents, and especially upon amalgam for dentists' use ever presented tot he dental profession.

As time advances experience in the manufacture and in the use of amalgam for filling carious teeth, for crown work, for repairing rubber or celluloid plates, etc., is assuming a magnitude of almost universal interest. The prejudices of the former many, and the obstinacy of the still remaining few who offer opposition to filling teeth with amalgam, leaves the discussion of this question fully in accord with legitimate professional honor.

Its use for a half century, attended during the time with marked improvements, fully vindicates its worth by the thousands of teeth saved and the warm gratitude of the possessors.

The making of a *good* alloy for filling teeth, is one requiring more skill, knowledge amd care than is usually imagined.

The essential properties, or rather characteristics, such as workable plasticity; suitable speed of crystalization; efficient hardness; non-shrinkage and non-expansion; of suitable color; will make good sub-marine work, and will not be too expensive, are qualities in number and value, that must strike any one capable of the comprehension of the subject, as not to be secured without much presistent labor and untiring thought.

There are three cardinal principles to govern in the making of a suitable alloy for dental amalgam.

1. Metals used should each have an affinity for mercury.

2. Those selected should be benign to tooth structure

3. The quality of expansion of some metals, and of contraction of others while being made into amalgam must be so regulated that the combination will harmonize these extremes so as to form a non-shrinkable and a non-expansible compound.

Silver, tin, gold, copper, zinc and some other metals readily unite with mercury, when they are in fine separation and some friction and presure is used. And each of these formed into an amalgam has a characteristic of its own.

The first four named enter into nearly all the best alloys found in the market; while the two first, forms much the largest ingredients in any dental alloy.

Tin in excess will lack hardness; will have a tendency to "ball" and draw away from the walls of the cavity. Silver in excess will contract and tend to leakage. Gold in excess will interfere with crystalization and strength. Copper, though invaluable in correcting contracting and securing the required edge strength, if in excess, will cause too much discoloration. Of zinc a very little is enough; and if properly managed greatly aids in preventing discoloration.

I cannot, from my experiments, find any good results from platinum in any quantity. Believe it will be found so, in an effort to use any metal which seems to have auch a disfavor with mercury. Probably in most cases the use of platinum in alloy is the result of a supposed or imaginable good; or in some a means to captivate trade. I am sorry I am unable to adduce a better reason. To call a compound by a name of an ingredient that forms but one one-hundredths or one four-hundredths part of its composition seems ridiculous. And even that little is useless: If useless, then how well that the quantity is infinitesimal.

The mere combination of metals while in a fused state does not always secure the best results obtainable from a good formula.

If in a four metal alloy a divison is made—two malts, and two ingots are made, each differing in quantities of its relative components, then cut separately and afterwards the two alloy fillings intimately mixed; it will be found that its properities when made into an amalgam is very different from that made where all the metals, of the same formula, were melted into one ingot and so cut and used. This after mixture of two alloys, each differing from the other, but in the aggregate being a same selected formula, produces a different molecular result, and opens a field for further experiments and better results.*

There is a strong tendency to use an amalgam that will not discolor in the mouth. In conspicious positions, of course, this may be well enough; but without doubt a degree of discoloration (some formation of sulphide salts) is favorable to tooth salvation. This dark sulphide of silver and copper is an insoluble salt that has the mechanical effect of preventing leakage, and the therapeutic effects of promoting recalcification of partially diseased dentine. In teeth of young subjects, and where frail walls, and where it is difficult to make retaining points—where a portion of carious dentine should remain undisturbed over a pulp, etc., this strong variety of amalgam that darkens some, and has more silver than tin, and not less than 4 per cent. copper, will do a very lasting and efficient service.

It has been proposed to use gold in combination with amalgam; building the gold upon the amalgam immediately after packing the latter. I believe in such work it is always better to let the amalgam become thoroughly hard before adding the gold; retaining points, etc., may then be made

---

*This after-mix has baffied seriously some of those who have found that the metals discovered in an alloy analyzis did not produce the same results when so compounded in the usual one melt plan. The attempt by the "Keller Medicine Company," of Fort Wayne, Ind., to reproduce any and all brands of alloy will be a miserable failure. In their card, No 10, they say they find in my "Old" 1 per cent. platinum and 8 per cent. zinc!!! and yet not a particle of either is put into it. They find *no copper* and yet it contains 5 *per cent. copper*. Such pretentions to analysis is simply outrageous. If even the formula was correctly known (and it's no secret) how could they produce the same if they melted all the metals at simply one fuse?

in the amalgam and tooth walls conjointly. In this way the gold will (as is generally desired) retain a better color, while all the benefit of compounding for the superiority of amalgam in cervical and frail marginal positions is as well secured. In compound cavities in bicuspids, and approximal cavities of cuspids and incisors, where the posterior or lingual borders are frail and must be freely cut away, this combination with gold and amalgam offers good and useful results, and makes clean artistic work without losing unnecessarily tooth tissue, or endangering a nearly exposed pulp. It has been observed that where gold has been worked into a fresh amalgam that even the amalgam gets darker than where used alone. This seems so; but if the amalgam is allowed first to harden before the gold is added this tendency is much, if not altogether, obviated.

For saving the deciduous teeth there is nothing in use that nearly equals amalgam; and I feel like urging the profession to consider its claims in this respect. No difference what the age of the child, or to what condition of society belonging, it offers the most good, the least suffering and fatigue to the little patient and also to the operator. How frequently the caries is just approaching the pulp chamber, and the warniugs of a restless night brings the case forth. Now to simply remove the caries well from the borders, disinfect (with a little carbolic acid) that overlying the pulp and fill at once with amalgam; (which can be done without the torture of the coffer dam; and even though the saliva may flow over the work while progressing,) a very good protection will result.

Right here permit me to say, in cases where temporary teeth are badly decayed in approximate surfaces, and from the short form of the teeth, and frailty of the walls difficulty attends the securing of a separate filling for each tooth; a very good way is to extend an amalgam filling from cavity to cavity. This joining of the teeth in such cases does admirably, and where they are a little apart, a small bar of silver can be laid with an end resting in each cavity and upon some already introduced alloy, and thus anchored with amalgam, each aids the other; and the bar has prevented the amalgum from being pressed unduly upon the intervening gums.

In the repair of a rubber or a celluloid plate, where a tooth or a block of teeth is to be restored, strong amalgam does it quickly and very completely. Grind and adjust the block, and directly beneath the pins cut holes and fissures with retaining walls. Fix the block in position by placing the edge of the gum border into a bed of warm gutta-percha, taking care that it does not interfere with the retaining orifices. Now pack the holes, and space about the pins, completely with amalgam, and when hard, dress up. If the amalgam is strong, and the case not put to use too soon, it will do excellent service. It is done quickly and saves the plate from the dangers and damage of reheating.

But this communication is getting too long, and with a word about testing amalgams I close.

Every operator should test his alloy in regard to all the indispensable characteristics.

His experience will soon qualify him to judge as to its suitable rate of crystalization. It may set too rapidly, or too slowly. He can judge pretty well of its quality of strength by testing with file, hammer, etc.

For expansion or contraction he should provide himself with small pieces (say half an inch in length) of glass test tubing (obtained at almost any drug store.) Fill some of these just as carefully as he would a cavity in a tooth. Place them in a solution of red analine; or in his writing ink. If the amalgam will contract, then the leakage will appear and can readily be seen. If it expands the glass tube will crack. In either case the alloy is faulty. For color test, a button of amalgam with a polished surface may be dropped into a solution of sulphuret of pottassa (forty grains to the ounce of water will do) and let remain twenty-four to seventy-two hours.

As before stated the whitest alloys should not be selected where the most strength and durability is needed. An amalgam that will darken but little in the above named solution may in most mouths remain quite sightly, and especially for molars be a very good grade, so far as color is concerned, and the best for durability.

Whatever amalgam is used in the mouth, every step requires and deserves being done well and skillfully. Each filling should be well evened up and polished after it has become thoroughly hard, and the necessary instructions imparted to the patient to keep them in clean and polished condition.—*Dental Luminary.*

